# Healthcare professionals’ sources of knowledge of complementary medicine in an academic center

**DOI:** 10.1371/journal.pone.0184979

**Published:** 2017-09-29

**Authors:** Eleonore Aveni, Brent Bauer, Anne-Sylvie Ramelet, Isabelle Decosterd, Pierluigi Ballabeni, Eric Bonvin, Pierre-Yves Rodondi

**Affiliations:** 1 Institute for Social and Preventive Medicine, Lausanne University Hospital, Lausanne, Switzerland; 2 Division of General Internal Medicine, Mayo Clinic, Rochester, Minnesota, United States of America; 3 Institute of Higher Education and Research in Healthcare-IUFRS, Lausanne University, Lausanne, Switzerland; 4 Pain Center, Department of Anesthesiology, Lausanne University Hospital, Lausanne, Switzerland; 5 Department of Psychiatry, Lausanne University Hospital, Lausanne, Switzerland; Partner Institute for Computational Biology Chinese Academy of Sciences and Max Planck Society, CHINA

## Abstract

**Background:**

Complementary medicine (CM) is utilized in a growing number of academic centers despite the debate concerning its value, risks and benefits. Healthcare professionals often feel uncomfortable discussing CM with patients, and little is known about their sources of knowledge in the field of CM.

**Objective:**

To assess healthcare professionals’ sources of knowledge and attitude toward CM in an academic hospital.

**Design and participants:**

The cross-sectional web-based survey took place from October to December 2013. A total of 4,925 healthcare professionals working at Lausanne University Hospital, Switzerland, were invited to answer the questionnaire.

**Main measures:**

Factors influencing healthcare professionals’ opinion toward CM, knowledge and communication about CM.

**Key results:**

The questionnaire was answered by 1,247 healthcare professionals. The three key factors influencing professionals’ opinion toward CM were personal experience, clinical experience and evidence demonstrating the physiological mechanism of CM. Personal experience was more associated with nurses’ and midwives’ opinion compared to physicians’ (80.8% vs 57.1%, OR = 3.08, [95% CI: 2.35–4.05], *P*<0.001 and 85.3% vs 57.1%, OR = 3.83, [95% CI: 1.95–7.53], *P*<0.001, respectively) as well as with professionals trained in CM compared to non-trained professionals (86.0% vs 73.2%, OR = 2.60, [95% CI: 1.92–3.53], *P*<0.001). Physicians relied more on randomized controlled clinical trials compared to nurses (81.3% vs 62.9%, OR = 0.43, [95% CI: 0.33–0.57], *P*<0.001). A majority of the respondents (82.5%) agreed that they lacked knowledge about CM and 65.0% noted that it was the patient who initially started the discussion about CM.

**Conclusions:**

Different professionals used different strategies to forge opinions regarding CM: physicians relied more on scientific evidence, while nurses and midwives were more influenced by personal experience. Regardless of preferred information source, most respondents did not feel prepared to address patient questions regarding CM. Enhancing interprofessional education opportunities is an important strategy to help providers become empowered to discuss CM with patients. This in turn will help patients making informed decisions in their healthcare.

## Introduction

The prevalence of complementary medicine (CM) use ranges between a quarter and a third of the population [1], with a rate of 25% in Switzerland [2]. In concordance with the patients’ demand [3] and the increasing interest of healthcare professionals [4–6], CM is being implemented in a growing number of academic centers [7] and education is progressively being incorporated into medical curricula [8]. Nevertheless, implementation of CM in hospitals is often weighed down by lack of policy, low rate of patient referral and medical skepticism [9]. The debate about the implementation of CM in hospitals is mostly based on the lack of both biomedical and clinical evidence for efficacy, lack of knowledge [10], along with concerns about safety [11]. Therefore, the legitimacy of CM in hospital setting is still contested [12].

Over the last century, progress in statistics and epidemiology have contributed to a massive development of clinical research [13]. Concurrently, Evidence-Based Medicine (EBM) and Evidence-Based Practice (EBP) describe an ideal process of making “clinical decisions based on the best available, current, valid and relevant evidence” [14]. In this context, knowledge translation has been defined as a “dynamic and iterative process […] of application of knowledge to improve health and provide more effective health services […]” [15]. Several studies have been carried out to describe the way healthcare professionals apply knowledge (from literature, research and personal experience) to practice [16] and some have revealed that healthcare professionals often felt overwhelmed by the flood of information [17].

Considering that CM might lead to interactions and side effects [18], it is of major importance for patients to disclose their use of CM to their physician, and for professionals to be able to inform patients adequately [19]. However, the range of non-disclosure of CM use by patients was reported between 23 and 70%, indicating that there is a need for better communication [20]. The American Association of Medical Colleges has emphasized the necessity for physicians to be “sufficiently knowledgeable about both traditional and non-traditional modes of care to provide intelligent guidance to their patients” [21]. Despite this statement and the interest of healthcare professionals discussed previously, most still declared feeling uncomfortable discussing CM with their patients [11] and lack of knowledge appeared to be the main reason for this reticence [22].

Regardless of the implementation of CM in academic centers, little is known about healthcare professionals’ knowledge, sources of information and ability to discuss CM with patients. The aim of this study was therefore to assess physicians’, nurses’, physical therapists’ and midwives’ knowledge and attitudes in the field of CM in an academic center.

## Methods

A cross-sectional survey was conducted among all physicians, nurses, physical therapists, and midwives working at the Lausanne University Hospital in Switzerland. This urban tertiary-care teaching hospital serves a population of 800,000 inhabitants of the state of Vaud. An invitation to complete an anonymous, web-based survey was e-mailed on October 8, 2013 to 4,925 healthcare professionals (3,196 females and 1,729 males), including 1,969 physicians, 2,697 nurses, 148 physical therapists, and 111 midwives. A reminder was sent one month later. In order to ease the recruitment, we offered every fifth respondent a voucher in a local bookshop.

We could not find any questionnaire that specifically investigated the way healthcare professionals’ were getting informed about CM. For this reason, and given the fact that previous studies had already used non-standardized questionnaires to investigate this topic, we decided to developed our own questionnaire, based on the following two previous studies [23,24]. A French and English version of our questionnaire can be found in the Supporting Information of this article and can be downloaded. The definition of complementary medicine used for this study was “a broad set of health care practices that are not part of that country's own tradition and are not integrated into the dominant health care system”, according to the World Health Organization [25]. We mainly worked with questionnaires in English, so we had to proceed to forward and backward translation (English-French), in order to ensure that the meaning of the questions was the same in both languages. The final version of the questionnaire was pre-tested by 10 healthcare professionals (2 physicians, 2 physical therapists and 6 nurses), chosen among the personal working in the academic hospital, in order to make final corrections and adjustments. The final questionnaire was then validated by all the authors of the present manuscript. It included 32 closed-ended questions and consisted of three parts: sociodemographic data, attitude toward the introduction of CM at an academic center for the treatment of chronic pain and general attitude toward CM. Questions related to attitudes and knowledge toward CM were rated using 5-point Likert-type scales. The fulfillment of the questionnaire took about 20 minutes. Results of the data about chronic pain were already published [26]. This manuscript presents the third part: “general attitude toward CM”, which investigated the association between several factors and professionals’ characteristics on their opinion toward CM. We also evaluated the way conversations with patients about CM were initiated. We explored knowledge about CM at the end of the questionnaire. There was no missing data in our survey as respondents had to answer every question in order to access the next question. Meanwhile, respondents had the possibility to check a “I do not know” or a “I do not want to answer this question” box. The study was accepted by the "Commission cantonale d'éthique de la recherche sur l'être humain".

We performed univariate logistic regression analysis. Attitudes and knowledge toward CM were analysed as dependent variables in models having gender or profession as explanatory variables. Since attitudes and knowledge towards CM where assessed by ordinal variables, ordered logistic regressions were used. Thus, odds ratios indicate how likely gender or professional categories were to answer at a higher attitude or knowledge compared to a reference category (males for gender; physicians for profession). Statistical significance was considered when p<0.05. Items with the “I do not know” answer were not included in the analyses. This is reflected by varying numbers of analysed observations in the results. Multivariate analysis was not performed. The statistical analyses were performed using Stata software, version 14.1 (StataCorp LP, TX, USA).

## Results

Of the 4,925 who were invited by email to answer the questionnaire, 1,247 responded (response rate = 25.3%). Of the respondents, 922 were women (73.9%, response rate = 28.8%) and 320 were men (25.7%, response rate = 18.5%). The breakdown by profession was as follows: 879 nurses (70.5%, response rate = 32.6%), 257 physicians (20.6%, response rate = 13.0%), 68 physical therapists (5.5%, response rate = 45.9%) and 34 midwives (2.7%, response rate = 30.6%). Gender, age categories, profession, main activity and training in CM are given in Table 1.

**Table 1:** Sociodemographics.

Sociodemographic characteristics of the respondents (N = 1,247)	N (%)
**Gender**	
Female	922 (73.9)
Male	320 (25.7)
NR	5 (0.4)
**Age**	
≤ 35 y	567 (45.5)
36–45 y	357 (28.6)
46–55 y	223 (17.9)
≥ 56 y	95 (7.6)
NR	5 (0.4)
**Profession**	
Nurse	879 (70.5)
Physician	257 (20.6)
Physical therapist	68 (5.5)
Midwife	34 (2.7)
NR	9 (0.7)
**Main activity**	
Clinic	1,072 (86.0)
Management	129 (10.3)
Research	46 (3.7)
**Trained in practicing one or more complementary medicine**	
Yes	197 (15.8)

NR: no response

Sociodemographic characteristics of the survey respondents.

The mean number of years of professional experience after graduation was 13.32 ± 9.85 SD (range 0–42, median: 11) and the mean number of years working at the university hospital was 8.82 ± 8.12 SD (range 0–41, median: 6). The majority of professionals were working directly with patients (n = 1,166, 93.5%). Of the 1,247 respondents, 197 (15.8%) were trained in a CM modality: 25 (9.7%) physicians, 148 (16.8%) nurses, 14 (20.6%) physical therapists, 9 (26.5%) midwives and 1 (0.1%) non-specified, with 59 (29.9%) trained in reflexology, 26 (13.2%) in aromatherapy, 25 (12.7%) in massage, and 23 (11.7%) in hypnosis. Gender distribution and professions of respondents were compared with non respondents in detail in another article and showed a good representativeness of our sample [26].

The three key factors influencing healthcare professionals’ opinion toward CM with major or high impact were personal experience (n = 885, 75.3%), clinical experience with patients (n = 774, 69.7%) and evidence demonstrating the physiological mechanism of CM treatments (n = 772, 69.3%), whereas case reports published in CM journal had the lowest impact (n = 383, 35.9%). The associations between several factors and professionals’ characteristics on their opinion toward CM are described in Table 2.

**Table 2:** Associations of several factors about complementary medicine with respondents’ characteristics, stratified by gender and profession.

	*Gender*		*Profession*			
	Male	Female	Physician	Nurse	Physical therapist	Midwife
		OR [95% CI] *P*		OR [95% CI] *P*	OR [95% CI] *P*	OR [95% CI] *P*
*Personal experience with positive results on myself*	1.00	2.66 [2.07–3.42]	1.00	3.08 [2.35–4.05]	1.50 [0.89–2.50]	3.83 [1.95–7.53]
		< 0.001		< 0.001	0.12	< 0.001
*Recommendations by family and friends who have tried the therapy*	1.00	3.58 [2.78–4.60]	1.00	3.90 [2.98–5.13]	1.75 [1.06–2.90]	7.59 [3.85–14.96]
		< 0.001		< 0.001	0.03	< 0.001
*Recommendations by colleagues who have tried the therapy themselves*	1.00	2.80 [2.19–3.60]	1.00	2.50 [1.90–3.27]	1.36 [0.83–2.24]	6.24 [3.15–12.36]
		< 0.001		< 0.001	0.23	< 0.001
*Recommendations by specialists or consultants to whom you have referred a patient*	1.00	2.11 [1.64–2.72]	1.00	1.70 [1.30–2.22]	1.13 [0.68–1.88]	4.32 [2.15–8.69]
		< 0.001		< 0.001	0.63	< 0.001
*Clinical experience in your patient population*	1.00	1.62 [1.27–2.08]	1.00	1.71 [1.31–2.23]	1.15 [0.69–1.93]	2.07 [1.06–4.04]
		< 0.001		< 0.001	0.59	0.03
*Post-graduate training / conferences*	1.00	1.71 [1.34–2.20]	1.00	1.97 [1.51–2.57]	1.38 [0.83–2.28]	2.2 [1.04–4.63]
		< 0.001		< 0.001	0.21	0.04
*Case report in CM journals*	1.00	2.03 [1.57–2.62]	1.00	3.11 [2.36–4.10]	2.08 [1.23–3.53]	7.95 [3.78–16.73]
		< 0.001		< 0.001	0.006	< 0.001
*Case report in standard medical journals*	1.00	1.31 [1.02–1.67]	1.00	1.50 [1.15-1-95]	1.29 [0.76–2.18]	3.43 [1.67–7.02]
		0.03		0.002	0.35	0.001
*Retrospective case–control studies reported in standard medical journals*	1.00	1.30 [1.01–1.67]	1.00	1.49 [1.14–1.95]	1.17 [0.70–1.95]	2.72 [1.35–5.47]
		0.04		0.003	0.55	0.005
*Prospective randomized controlled clinical trials published in medical journals*	1.00	0.62 [0.48–0.80]	1.00	0.43 [0.33–0.57]	0.50 [0.30–0.85]	0.86 [0.43–1.69]
		< 0.001		< 0.001	0.01	0.66
*Evidence demonstrating the physiological mechanism of CM treatments*	1.00	1.00 [0.78–1.28]	1.00	0.99 [0.76–1.29]	0.87 [0.52–1.47]	1.10 [0.55–2.10]
		0.99		0.93	0.61	0.80
*Guidelines*	1.00	1.20 [0.94–1.54]	1.00	0.96 [0.74–1.25]	0.84 [0.51–1.39]	2.09 [0.97-4-50]
		0.14		0.76	0.50	0.06

CM: complementary medicine.

Associations of several factors about complementary medicine with respondents’ characteristics, stratified by gender and profession. Results for “major impact” and “high impact” on professionals’ opinion toward complementary medicine are presented in this table.

Comparison between CM trained and non-trained healthcare professionals showed that trained professionals gave higher influence to suggested factors (both experience-based and literature-based factors) compared to non-trained professionals. Details are given in Table 3.

**Table 3:** Association of several factors with trained vs. non-trained healthcare professionals.

	Trained professionals
	OR [95% CI] *P*
*Personal experience with positive results on myself*	2.60 [1.92–3.53]
	< 0.001
*Recommendations by family and friends who have tried the therapy*	1.62 [1.22–2.15]
	0.001
*Recommendations by specialists or consultants to whom you have referred a patient*	1.51 [1.14–2.00]
	0.005
*Recommendations by colleagues who have tried the therapy themselves*	1.70 [1.25–2.30]
	0.001
*Clinical experience in your patient population*	1.71 [1.27–2.29]
	< 0.001
*Post-graduate formation / conferences*	2.23 [1.66–2.99]
	< 0.001
*Case report in complementary medicine journals*	2.22 [1.65–2.97]
	< 0.001
*Case report in standard medical journals*	1.69 [1.26–2.27]
	< 0.001
*Retrospective case–control studies reported in standard medical journals*	1.59 [1.19–2.13]
	0.002
*Prospective randomized controlled clinical trials published in medical journals*	1.24 [0.92–1.67]
	0.15
*Evidence demonstrating the physiological mechanism of complementary medicine treatments*	1.37 [1.03–1.83]
	0.03
*Guidelines*	1.32 [0.98–1.78]
	0.07

Association between several factors and trained vs non-trained healthcare professionals. Are presented here the results for “major impact” and “high impact” on professionals’ opinion toward complementary medicine. The odd ratios and *P* values correspond to trained professionals compared to non-trained healthcare professionals.

Analyses by age categories and main activity (clinical practice, management, research) showed no statistical difference.

When asked about their knowledge about CM, a majority of the respondents (n = 1,029, 82.5%) agreed or strongly agreed that they lacked knowledge about CM. However, 1,047 (84.0%) thought that healthcare professionals should have knowledge about the most prominent CM treatments and 1,040 (84.0%) approved that healthcare professionals should be able to inform patients about CM.

More than half of the respondents (n = 603, 65.0%) noted that it was the patient who initially started the discussion about CM. Females were more likely to initiate than males (37.3% vs 28.2%, OR = 1.51, [95% CI: 1.10–2.09], *P* = 0.01), and nurses and midwives more likely to initiate than physicians (35.6% vs 26.9%, OR = 1.50, [95% CI: 1.06–2.14], *P* = 0.02 and 66.7% vs 26.9%, OR = 5.44, [95% CI: 2.48–11.97], *P*<0.001, respectively). Finally, training in CM was associated with a higher rate of initiation of discussion (44.6% vs 32.9%, OR = 1.64, [95% CI: 1.16–2.30], *P* = 0.005).

## Discussion

We observed that among the factors influencing healthcare professionals’ attitudes toward CM in a Swiss academic hospital, personal experience, recommendations by other people (both healthcare professionals or not), and clinical experience with patients were more strongly associated with nurses’, midwives’ and females’ attitudes compared to physicians’ and males’ attitudes, respectively. Opinions of physicians and males about CM were more related to results of randomized controlled trials. In a US study among physicians, “results of randomized controlled trials” and “evidence demonstrating the treatment’s mechanism” were the only two factors identified by more than half of the physicians to have a high or definite impact on their attitude toward CM [5]. The influence of literature on physicians’ opinion, at the expense of their own experience, has already been discussed in previous articles. Indeed, Silverman established that medical training, while focusing on biomedical and scientific aspects, was directly affecting communication with patients and that, by the end of residency, many physicians distanced themselves from the human perspective of medicine [27]. Furthermore, lack of time and knowledge appeared to be among the main reasons hindering the implementation of medical knowledge into physicians’ practice [28]. Similarly to our study, personal experience was an essential source of information among nurses, as they rather used informal and interactive sources to get informed [29]. The gap between theory and practice in nurses’ practice has already been discussed in articles [30], and revealed that student nurses usually do not intend to research evidence in the literature [31], although understanding the importance of EBM and research [32]. Furthermore, a study revealed that EBP was lacking in nurses’ daily practice [33]. In spite of the development of EBP and the evidence for its efficacy [34], nurses’ practice is still mainly based on intuition, tradition and experience [35]. Hatlevik suggested that reflective skills and theoretical knowledge were essential in order to bridging the theory-practice gap [36]. Similarly to nurses, physical therapists, although understanding and demonstrating good knowledge on EBP and literature research, did not regularly engage in the steps of EBP [37]. They would rather use social interactions as information sources. To our knowledge, there is no study describing the knowledge translation process among midwives.

In our study, two thirds of the discussions about CM were initiated by patients. A vast majority of respondents affirmed that they lacked knowledge about CM, although agreeing that they should be aware of the most prominent CM and that they should therefore be able to inform their patients. Lack of knowledge about CM concerned up to 83.6% of the nurses in our study, which is higher than in another study among oncology nurses, where about a fifth lacked knowledge about CM [38]. This difference could be linked with environmental factors; as the use of CM is higher among oncology patients, oncology nurses might be more in contact with CM, and thus feel more knowledgeable about it. The lack of communication and the unease of healthcare professionals discussing CM has already been described [11], with females, CM users and knowledgeable professionals being more likely to recommend CM to patients [39]. These results correlate with ours, that female and CM trained professionals were more likely to start a discussion about CM. The lack of communication between patients and healthcare providers is an important issue. Patients mentioned the physicians’ indifference or opposition to CM and the fact that the latter did not ask as the main reasons for not disclosing the use of CM [20]. Therefore, patients often manage the interface between CM and conventional medicine on their own. Considering these results and the fact that the rate of discrepancies between physicians’ and CM practitioners’ advice can be as high as ten percent [40], finding ways to promote knowledge and discussions about CM is of major importance. First, interprofessional education, which involves different professionals learning about, with and from each other’s [41], might be a pathway to increasing healthcare professionals’ knowledge of CM. In fact, interprofessional education is increasingly being introduced in medical schools [42], even in the CM field [43,44], as it has been demonstrated to lead to fewer medical errors, resulting therefore in better outcomes [45,46]. Furthermore, interprofessional education was established to be a significant strategy for reducing the bias between conventional medicine and CM providers [47] and a recent pilot study involving students from several professions concluded that interprofessional education was leading to increased awareness and knowledge toward CM, promoting therefore coordinated care [48]. Secondly, professionals must be encouraged to initiate conversation about CM with patients, independently of the backgrounds or complaints of the latter.

There are some limitations to this study. First, our questionnaire was built using questions from other surveys, as no validated questionnaire existed on this topic; it is thus not a validated form. Second, the survey has been held in only one academic hospital in Switzerland. The results in other hospitals might be different. Furthermore, we cannot generalize the results to all healthcare professionals in our hospital, as the response rate was quite low. Lack of interest for complementary medicine among healthcare professionals could be an explanation, as perceived salience of a study has been described as a factor linked with low response rate among physicians [49]. Another reason could be lack of time of healthcare professionals, especially linked with the length of the questionnaire [50]. Third, professionals interested in CM could have participated more than others. Meanwhile, to decrease this potential bias, we noted on the advertisement that taking part in the study was a good opportunity to give one’s opinion.

## Conclusions

Our results showed a trend in the way healthcare professionals are getting informed and forge their opinion toward CM: physicians tended to rely more on scientific evidence and literature, while nurses and midwives relied more on personal and clinical experience. With the documented reluctance of patients to disclose their use of CM, professionals must take a more active role in initiating discussions about CM. In parallel, healthcare professionals must also have a better foundation of knowledge about CM. Given the rapidly evolving research in this area, there is a need for good quality information about CM to be easily available at the hospital, and interprofessional collaboration should be taken into account as a mean to getting to it.

## Supporting information

S1 File English-French questionnaire. (DOCX)
