# Supplementary material for: Healthcare professionals’ sources of knowledge of complementary medicine in an academic center
Source: PLoS One. 2017 Sep 29;12(9):e0184979. doi: 10.1371/journal.pone.0184979 (PMC5621686; doi:10.1371/journal.pone.0184979)
Supplement: S1 File — (DOCX) [file pone.0184979.s001.docx]

| **Questionnaire à l’intention des médecins et infirmiers du CHUV**  Le traitement de la douleur fait partie des directives institutionnelles du CHUV. De nombreux patients souffrant de douleurs chroniques ont recours à des thérapies complémentaires. Nous souhaitons connaître votre opinion sur les médecines complémentaires dans le traitement des douleurs chroniques.  Il faut compter au maximum 10-15 minutes pour remplir le questionnaire.  Définitions utilisées dans le cadre de cette étude :   - Douleur chronique : douleur ressentie par le patient depuis > 3 mois. - Médecines complémentaires (OMS) : un large ensemble de pratiques de soins qui ne font pas partie de la tradition (académique) du pays ou qui ne sont pas intégrées dans le système de santé dominant (par exemple en Suisse, l’acupuncture, l’ostéopathie, l’homéopathie…). - Dans ce questionnaire, certaines thérapies comme l’hypnose ou la musicothérapie sont considérées comme des médecines complémentaires car elles ne sont pas intégrées de manière systématique dans le système de santé en Suisse.   Ce questionnaire est réalisé dans le cadre d’un travail de master en médecine. Pour toute question, vous pouvez vous adresser à [Eleonore.aveni@unil.ch](mailto:Eleonore.aveni@unil.ch)  Toutes les réponses seront traitées de manière anonyme.  Nous vous remercions de répondre aux questions suivantes :  ***Données socio-démographiques :***   - Genre : F H - Tranche d’âge :   - ≤ 35 ans   - 36-45 ans   - 46-55 ans   - ≥ 56 ans   - Ne souhaite pas répondre - Ethnie :   - Europe   - Afrique   - Amérique centrale et du sud   - Amérique du nord   - Asie   - Océanie   - Inconnu / apatride   - Ne souhaite pas répondre - Profession :   Médecin  Infirmier/infirmière  Physiothérapeute  Sage-femme  Autre / ne souhaite pas répondre   - Fonction (pour les médecins) :   - Médecin-assistant   - Chef de clinique   - Médecin-cadre - Pour les infirmiers/ères :   - Je possède une spécialisation (clinicien/ne, salle d’opération, SI, anesthésie, urgences, practicien/ne-formateur)   - Je travaille comme ICUS / ICS - Pour les infirmiers/ères : quelle est votre formation post-grade la plus élevée ? - Pour les physiothérapeutes, je travaille comme PCU / PCS :   - Oui   - Non - Pour les sages-femmes :   - Je possède une spécialisation (ICL, PF)   - Je travaille comme ICUS / ICS - Nombre d’années d’expérience professionnelle après l’obtention de votre diplôme : - Depuis combien de temps travaillez-vous au CHUV ? ­­­­­­­­­­­ - Quelle est la part la plus importante de votre activité :   - Clinique   - Gestion   - Recherche - Etes-vous en contact direct avec des patients ? - La prise en charge des douleurs chroniques fait-elle partie de votre rôle professionnel ? - Dans quel Département travaillez-vous ?   - Département de médecine (DM)   - Département des services de chirurgie et d’anesthésiologie (DSCA)   - Département de gynécologie-obstétrique et génétique (DGOG)   - Département médico-chirurgical de pédiatrie (DMCP)   - Département universitaire de médecine et santé communautaires (DUMSC)   - Département de l’appareil locomoteur (DAL)   - Département des centres interdisciplinaires et logistique médicale (DCILM)   - Département des Neurosciences Cliniques (DNC)   - Département de psychiatrie (DP)   - Département d’oncologie (DO)   - Département formation et recherche (DFR)   - Département des laboratoires (DL)   - Département de radiologie médicale (DRM)   - Autre département   - Ne souhaite pas répondre - Avez-vous suivi une formation dans la pratique d’une (ou plusieurs) médecine(s) complémentaire(s) :   - Oui     - Si oui, laquelle/lesquelles ?   - Non   ***Attitude face à l’introduction de certaines médecines complémentaires dans un centre universitaire pour le traitement de la douleur chronique :***   - Pensez-vous que certaines médecines complémentaires puissent se révéler utiles dans le traitement de patients avec des douleurs chroniques ?   - Tout à fait d’accord   - D’accord   - Ni d’accord, ni pas d’accord   - Pas d’accord   - Pas du tout d’accord - Recommanderiez-vous à un patient souffrant de douleurs chroniques de recourir à une médecine complémentaire si celle-ci était proposée au CHUV ? - Tout à fait d’accord   - D’accord   - Ni d’accord, ni pas d’accord   - Pas d’accord   - Pas du tout d’accord - Avez-vous déjà référé un patient à un thérapeute pratiquant une médecine complémentaire pour le traitement de douleurs chroniques ?   - Oui   - Non - Etes-vous favorable à l’introduction au CHUV (pas forcément dans le service où vous travaillez) de certaines médecines complémentaires dont l’efficacité a été prouvée dans le traitement de la douleur chronique ?   - Oui, très favorable   - Oui, favorable   - Ni oui, ni non (position neutre)   - Non, pas favorable   - Non, pas du tout favorable   Etes-vous favorable à l’introduction au CHUV (pas forcément dans le service où vous travaillez) des médecines complémentaires suivantes ?   - - Acupuncture     - Oui, très favorable     - Oui, favorable     - Ni oui, ni non (position neutre)     - Non, pas favorable     - Non, pas du tout favorable     - Je ne connais pas cette thérapie   - Aromathérapie / huiles essentielles     - Oui, très favorable     - Oui, favorable     - Ni oui, ni non (position neutre)     - Non, pas favorable     - Non, pas du tout favorable     - Je ne connais pas cette thérapie   - Art-thérapie     - Oui, très favorable     - Oui, favorable     - Ni oui, ni non (position neutre)     - Non, pas favorable     - Non, pas du tout favorable     - Je ne connais pas cette thérapie   - Biofeedback     - Oui, très favorable     - Oui, favorable     - Ni oui, ni non (position neutre)     - Non, pas favorable     - Non, pas du tout favorable     - Je ne connais pas cette thérapie   - Hypnose     - Oui, très favorable     - Oui, favorable     - Ni oui, ni non (position neutre)     - Non, pas favorable     - Non, pas du tout favorable     - Je ne connais pas cette thérapie   - Homéopathie     - Oui, très favorable     - Oui, favorable     - Ni oui, ni non (position neutre)     - Non, pas favorable     - Non, pas du tout favorable     - Je ne connais pas cette thérapie   - Médecine anthroposophique     - Oui, très favorable     - Oui, favorable     - Ni oui, ni non (position neutre)     - Non, pas favorable     - Non, pas du tout favorable     - Je ne connais pas cette thérapie   - Médecine ayurvédique     - Oui, très favorable     - Oui, favorable     - Ni oui, ni non (position neutre)     - Non, pas favorable     - Non, pas du tout favorable     - Je ne connais pas cette thérapie   - Médecine traditionnelle chinoise     - Oui, très favorable     - Oui, favorable     - Ni oui, ni non (position neutre)     - Non, pas favorable     - Non, pas du tout favorable     - Je ne connais pas cette thérapie   - Méditation     - Oui, très favorable     - Oui, favorable     - Ni oui, ni non (position neutre)     - Non, pas favorable     - Non, pas du tout favorable     - Je ne connais pas cette thérapie   - Mindfulness based stress reduction (MBSR)     - Oui, très favorable     - Oui, favorable     - Ni oui, ni non (position neutre)     - Non, pas favorable     - Non, pas du tout favorable     - Je ne connais pas cette thérapie   - Musicothérapie     - Oui, très favorable     - Oui, favorable     - Ni oui, ni non (position neutre)     - Non, pas favorable     - Non, pas du tout favorable     - Je ne connais pas cette thérapie   - Naturopathie     - Oui, très favorable     - Oui, favorable     - Ni oui, ni non (position neutre)     - Non, pas favorable     - Non, pas du tout favorable     - Je ne connais pas cette thérapie   - Ostéopathie     - Oui, très favorable     - Oui, favorable     - Ni oui, ni non (position neutre)     - Non, pas favorable     - Non, pas du tout favorable     - Je ne connais pas cette thérapie   - Phytothérapie     - Oui, très favorable     - Oui, favorable     - Ni oui, ni non (position neutre)     - Non, pas favorable     - Non, pas du tout favorable     - Je ne connais pas cette thérapie   - Reiki     - Oui, très favorable     - Oui, favorable     - Ni oui, ni non (position neutre)     - Non, pas favorable     - Non, pas du tout favorable     - Je ne connais pas cette thérapie   - Réflexothérapie     - Oui, très favorable     - Oui, favorable     - Ni oui, ni non (position neutre)     - Non, pas favorable     - Non, pas du tout favorable     - Je ne connais pas cette thérapie   - Tai chi     - Oui, très favorable     - Oui, favorable     - Ni oui, ni non (position neutre)     - Non, pas favorable     - Non, pas du tout favorable     - Je ne connais pas cette thérapie   - Thérapie neurale     - Oui, très favorable     - Oui, favorable     - Ni oui, ni non (position neutre)     - Non, pas favorable     - Non, pas du tout favorable     - Je ne connais pas cette thérapie   - Yoga     - Oui, très favorable     - Oui, favorable     - Ni oui, ni non (position neutre)     - Non, pas favorable     - Non, pas du tout favorable     - Je ne connais pas cette thérapie - Je recommanderais un traitement par acupuncture en complément à une prise en charge globale à un patient souffrant de :   - Céphalées de tension     - Tout à fait d’accord     - D’accord     - Ni d’accord, ni pas d’accord     - Pas d’accord     - Pas du tout d’accord     - Ne sait pas   - Cervicalgies     - Tout à fait d’accord     - D’accord     - Ni d’accord, ni pas d’accord     - Pas d’accord     - Pas du tout d’accord     - Ne sait pas   - Douleurs abdominales     - Tout à fait d’accord     - D’accord     - Ni d’accord, ni pas d’accord     - Pas d’accord     - Pas du tout d’accord     - Ne sait pas   - Douleurs chroniques dans un contexte oncologique (par exemple des métastases osseuses)     - Tout à fait d’accord     - D’accord     - Ni d’accord, ni pas d’accord     - Pas d’accord     - Pas du tout d’accord     - Ne sait pas   - Douleurs neuropathiques     - Tout à fait d’accord     - D’accord     - Ni d’accord, ni pas d’accord     - Pas d’accord     - Pas du tout d’accord     - Ne sait pas   - Douleurs pelviennes chroniques     - Tout à fait d’accord     - D’accord     - Ni d’accord, ni pas d’accord     - Pas d’accord     - Pas du tout d’accord     - Ne sait pas   - Douleur post-herpétique     - Tout à fait d’accord     - D’accord     - Ni d’accord, ni pas d’accord     - Pas d’accord     - Pas du tout d’accord     - Ne sait pas   - Fibromyalgie     - Tout à fait d’accord     - D’accord     - Ni d’accord, ni pas d’accord     - Pas d’accord     - Pas du tout d’accord     - Ne sait pas   - Lombalgies     - Tout à fait d’accord     - D’accord     - Ni d’accord, ni pas d’accord     - Pas d’accord     - Pas du tout d’accord     - Ne sait pas   - Migraines     - Tout à fait d’accord     - D’accord     - Ni d’accord, ni pas d’accord     - Pas d’accord     - Pas du tout d’accord     - Ne sait pas   ***Attitude face aux médecines complémentaires en général (pas uniquement dans le cas des douleurs chroniques)***   - Pour vous forger une opinion sur l’efficacité d’une médecine complémentaire, quel est l’impact de ces différents facteurs ?   - Expérience personnelle avec des résultats positifs sur moi-même     - Impact majeur     - Impact élevé     - Impact modéré     - Impact minime     - Pas d’impact     - Ne sait pas   - Recommandations d’amis/famille qui ont essayé une thérapie complémentaire     - Impact majeur     - Impact élevé     - Impact modéré     - Impact minime     - Pas d’impact     - Ne sait pas   - Recommandations de collègues qui ont essayé une thérapie  complémentaire     - Impact majeur     - Impact élevé     - Impact modéré     - Impact minime     - Pas d’impact     - Ne sait pas   - Recommandations de collègues spécialistes ou consultants à qui vous avez référé un patient     - Impact majeur     - Impact élevé     - Impact modéré     - Impact minime     - Pas d’impact     - Ne sait pas   - Expérience clinique de patients que vous avez suivis     - Impact majeur     - Impact élevé     - Impact modéré     - Impact minime     - Pas d’impact     - Ne sait pas   - Case reports (cas de patients) publiés dans des journaux de médecines complémentaires     - Impact majeur     - Impact élevé     - Impact modéré     - Impact minime     - Pas d’impact     - Ne sait pas   - Case reports (cas de patients) publiés dans des journaux médicaux conventionnels     - Impact majeur     - Impact élevé     - Impact modéré     - Impact minime     - Pas d’impact     - Ne sait pas   - Etudes rétrospectives publiées dans des journaux médicaux     - Impact majeur     - Impact élevé     - Impact modéré     - Impact minime     - Pas d’impact     - Ne sait pas   - Etudes randomisées contrôlées prospectives publiées dans des journaux médicaux     - Impact majeur     - Impact élevé     - Impact modéré     - Impact minime     - Pas d’impact     - Ne sait pas   - Preuves démontrant le mécanisme physiologique d’une médecine complémentaire     - Impact majeur     - Impact élevé     - Impact modéré     - Impact minime     - Pas d’impact     - Ne sait pas   - Cours / formation post-graduée / congrès     - Impact majeur     - Impact élevé     - Impact modéré     - Impact minime     - Pas d’impact     - Ne sait pas   - Recommandations de pratique clinique (guidelines)     - Impact majeur     - Impact élevé     - Impact modéré     - Impact minime     - Pas d’impact     - Ne sait pas - Lors d’une conversation avec un patient à propos de médecines complémentaires, qui initie la conversation en général ?   - Vous   - Le patient   - Ne s’applique pas - A quel pourcentage de vos patients parlez-vous des bénéfices des médecines complémentaires ?   - 0%   - 1-25%   - 26-50%   - 51-75%   - 76-99%   - 100% - A quel pourcentage de vos patients parlez-vous des risques des médecines complémentaires ?   - 0%   - 1-25%   - 26-50%   - 51-75%   - 76-99%   - 100% - Au cas où les preuves scientifiques de l’efficacité d’une médecine complémentaire dans une indication étaient disponibles, la recommanderiez-vous à vos patients ?   - Oui, très favorable   - Oui, favorable   - Ni oui, ni non (avis neutre)   - Non, pas favorable   - Non, pas du tout favorable - Pensez-vous que l’introduction de certaines médecines complémentaires dans un centre universitaire puisse avoir un impact sur le niveau de satisfaction des patients ?   - Oui, un impact très positif   - Oui, un impact positif   - Aucun impact   - Oui, un impact négatif   - Oui, un impact très négatif - Pensez-vous que l’introduction de certaines médecines complémentaires au CHUV puisse attirer plus de patients ?   - Tout à fait d’accord   - D’accord   - Ni d’accord, ni pas d’accord   - Pas d’accord   - Pas du tout d’accord - Pensez-vous que vos connaissances sont suffisantes pour renseigner vos patients au sujet des médecines complémentaires ?   - Tout à fait d’accord   - D’accord   - Ni d’accord, ni pas d’accord   - Pas d’accord   - Pas du tout d’accord - Avec quelle facilité pouvez-vous trouver des informations fiables au CHUV concernant l’usage des plantes médicinales ou de la phytothérapie (indications, contre-indications, effets indésirables, interactions…) ?   - Très facilement   - Facilement   - Ni facilement, ni difficilement   - Difficilement   - Très difficilement   - Je ne sais pas - Avec quelle facilité pouvez-vous trouver des informations fiables au CHUV concernant d’autres médecines complémentaires (indications, contre-indications, effets indésirables, interactions…) ?   - Très facilement   - Facilement   - Ni facilement, ni difficilement   - Difficilement   - Très difficilement   - Je ne sais pas - Que pensez-vous des affirmations suivantes ?  1. Les patients dont le médecin / l’infirmier/ère / le physiothérapeute / la sage-femme est formé et pratique avec eux une/des médecine(s) complémentaire(s) en plus de la médecine conventionnelle ont de meilleurs résultats cliniques que ceux dont le médecin ne pratique que la médecine conventionnelle.  - Désapprouve fortement - Désapprouve - Ni l’un, ni l’autre - Approuve - Approuve fortement  1. Les croyances et pratiques spirituelles des médecins / infirmiers/ères / physiothérapeutes / sages-femmes jouent un rôle dans l’état de santé des patients.  - Désapprouve fortement - Désapprouve - Ni l’un, ni l’autre - Approuve - Approuve fortement  1. Les croyances et pratiques spirituelles des patients jouent un rôle dans leur état de santé.  - Désapprouve fortement - Désapprouve - Ni l’un, ni l’autre - Approuve - Approuve fortement  1. Les médecins / infirmiers/ères / physiothérapeutes / sages-femmes devraient avoir des connaissances de base sur les médecines complémentaires les plus connues.  - Désapprouve fortement - Désapprouve - Ni l’un, ni l’autre - Approuve - Approuve fortement  1. Je pense que les traitements par les médecines complémentaires ont un véritable effet sur certains symptômes et/ou maladies.  - Désapprouve fortement - Désapprouve - Ni l’un, ni l’autre - Approuve - Approuve fortement  1. Je pense que le CHUV devrait proposer des thérapies complémentaires dont l’efficacité a été démontrée scientifiquement.  - Désapprouve fortement - Désapprouve - Ni l’un, ni l’autre - Approuve - Approuve fortement  1. L’introduction des médecines complémentaires pourrait porter une atteinte négative à la réputation du CHUV :    - Tout à fait d’accord    - D’accord    - Ni d’accord, pas d’accord    - Pas d’accord    - Pas du tout d’accord 2. Les médecines complémentaires offrent un bon rapport efficacité/coût.    - Tout à fait d’accord    - D’accord    - Ni d’accord, ni pas d’accord    - Pas d’accord    - Pas du tout d’accord 3. Les professionnels de santé devraient pouvoir informer les patients au sujet des médecines complémentaires.    - Tout à fait d’accord    - D’accord    - Ni d’accord, ni pas d’accord    - Pas d’accord    - Pas du tout d’accord 4. Il est nécessaire de développer davantage de recherches scientifiques sur les médecines complémentaires.    - Tout à fait d’accord    - D’accord    - Ni d’accord, ni pas d’accord    - Pas d’accord    - Pas du tout d’accord 5. Je manque d’informations au sujet des médecines complémentaires.    - Tout à fait d’accord    - D’accord    - Ni d’accord, ni pas d’accord    - Pas d’accord    - Pas du tout d’accord   Avez-vous des attentes particulières de la part du CHUV dans le domaine des médecines complémentaires ?  Avez-vous des commentaires sur les médecines complémentaires ?  Avez-vous des commentaires sur le questionnaire ? | **Questionnaire for the CHUV’s physicians and nurses**  The treatment of pain is part of the institutional directives of the CHUV. Numerous patients suffering from chronic pain resort to complementary therapies. We would like to know your opinion about complementary medicines in the treatment of chronic pain.  This questionnaire will take a maximum of 10-15 minutes to complete.  Definitions used for this survey:   - Chronic pain: pain felt by the patient for more than 3 months. - Complementary medicines = CM (OMS definition): a broad set of health care practices that are not part of that country's own tradition and are not integrated into the dominant health care system (for example, in Switzerland, acupuncture, osteopathy, homeopathy…).   In this questionnaire, some therapies such as hypnosis or music therapy are considered to be complementary medicines because they are not systematically integrated into the Swiss healthcare system.  This questionnaire is part of a Master’s degree in medicine. If you have any questions, you can contact [Eleonore.aveni@unil.ch](mailto:Eleonore.aveni@unil.ch)  All answers will be treated anonymously.  We thank you for answering the following questions:  ***Socio-demographic data :***   - Gender : F M - Age category :   - ≤ 35 years old   - 36-45 years old   - 46-55 years old   - ≥ 56 years old   - Do not wish to answer - Ethnicity :   - European   - African   - Central / south American   - North Amercian   - Asian   - Oceanian   - Unknown   - Do not wish to answer - Profession :   Physician - doctor  Nurse  Physical therapist  Midwife  Other / Do not wish to answer   - Function (for physicians):   - Foundation/resident   - Registrar/fellow   - Consultant - For nurses:   - I have a specialisation   - I am working as chief nurse - For nurses: what is your highest post-graduate formation (CAS, DAS...)? - For physical therapists: I am working as chief physical therapist?   - Yes   - No - For midwives:   - I have a specialisation   - I am working as chief midwife - Years of professional experience after graduation: - How long have you been working in the CHUV? - What is your main activity:   - Clinic   - Administration   - Research - Are you in contact with patients? - Are you currently taking care of patients suffering from chronic pain? - In which Department are your working?   - Department of general medicine   - Department of surgery and anaesthesiology   - Department of gynaecology and obstetrics   - Department of paediatrics   - Department of public health   - Department of locomotive apparatus   - Department of medical logistics   - Department of clinical neurosciences   - Department of psychiatry   - Department of oncology   - Department of formation and research   - Department of laboratory   - Department of medical imaging   - Other   - Do not wish to answer - Do you have training in practicing a complementary medicine?   - Yes     - Please, specify:   - No   ***Attitude towards the introduction of some complementary medicines in an academic centre in the treatment of chronic pain:***   - Do you think that some CM modalities can be useful for the treatment of patients with chronic pain?   - Totally agree   - Agree   - Neither agree nor disagree   - Disagree   - Totally disagree - Would you recommend complementary medicine to a patient with chronic pain if it was available at the CHUV?   - Totally agree   - Agree   - Disagree   - Totally disagree   - Neither agree nor disagree - Have you ever referred a patient to a CM practitioner for the treatment of chronic pain?   - Yes   - No - Are you in favour of the introduction (not necessarily in the service where you work) of some CM that have been proven efficient in the treatment of chronic pain in the CHUV?   - Yes, very favourable   - Yes, favourable   - Neutral   - No, not favourable   - No, not favourable at all   The same question applies to each of the following therapies:   - - Acupuncture     - Yes, very favourable     - Yes, favourable     - Neutral     - No, not favourable     - No, not favourable at all     - I do not know this therapy   - Aromatherapy / essential oils     - Yes, very favourable     - Yes, favourable     - Neutral     - No, not favourable     - No, not favourable at all     - I do not know this therapy   - Art-therapy     - Yes, very favourable     - Yes, favourable     - Neutral     - No, not favourable     - No, not favourable at all     - I do not know this therapy   - Biofeedback     - Yes, very favourable     - Yes, favourable     - Neutral     - No, not favourable     - No, not favourable at all     - I do not know this therapy   - Hypnosis     - Yes, very favourable     - Yes, favourable     - Neutral     - No, not favourable     - No, not favourable at all     - I do not know this therapy   - Homeopathy     - Yes, very favourable     - Yes, favourable     - Neutral     - No, not favourable     - No, not favourable at all     - I do not know this therapy   - Anthroposophic medicine     - Yes, very favourable     - Yes, favourable     - Neutral     - No, not favourable     - No, not favourable at all     - I do not know this therapy   - Ayurvedic medicine     - Yes, very favourable     - Yes, favourable     - Neutral     - No, not favourable     - No, not favourable at all     - I do not know this therapy   - Traditional Chinese Medicine     - Yes, very favourable     - Yes, favourable     - Neutral     - No, not favourable     - No, not favourable at all     - I do not know this therapy   - Meditation     - Yes, very favourable     - Yes, favourable     - Neutral     - No, not favourable     - No, not favourable at all     - I do not know this therapy   - Mindfulness based stress reduction (MBSR)     - Yes, very favourable     - Yes, favourable     - Neutral     - No, not favourable     - No, not favourable at all     - I do not know this therapy   - Music therapy     - Yes, very favourable     - Yes, favourable     - Neutral     - No, not favourable     - No, not favourable at all     - I do not know this therapy   - Naturopathy     - Yes, very favourable     - Yes, favourable     - Neutral     - No, not favourable     - No, not favourable at all     - I do not know this therapy   - Osteopathy     - Yes, very favourable     - Yes, favourable     - Neutral     - No, not favourable     - No, not favourable at all     - I do not know this therapy   - Herbal medicine     - Yes, very favourable     - Yes, favourable     - Neutral     - No, not favourable     - No, not favourable at all     - I do not know this therapy   - Reiki     - Yes, very favourable     - Yes, favourable     - Neutral     - No, not favourable     - No, not favourable at all     - I do not know this therapy   - Reflexotherapy     - Yes, very favourable     - Yes, favourable     - Neutral     - No, not favourable     - No, not favourable at all     - I do not know this therapy   - Tai chi     - Yes, very favourable     - Yes, favourable     - Neutral     - No, not favourable     - No, not favourable at all     - I do not know this therapy   - Neural therapy     - Yes, very favourable     - Yes, favourable     - Neutral     - No, not favourable     - No, not favourable at all     - I do not know this therapy   - Yoga     - Yes, very favourable     - Yes, favourable     - Neutral     - No, not favourable     - No, not favourable at all     - I do not know this therapy - About the use of acupuncture in chronic pain, I would refer a patient with chronic pain for an acupuncture treatment:   - Tension headaches     - Totally agree     - Agree     - Nor agree nor disagree     - Disagree     - Totally disagree     - I do not know   - Cervicalgia     - Totally agree     - Agree     - Nor agree nor disagree     - Disagree     - Totally disagree     - I do not know   - Abdominal pain     - Totally agree     - Agree     - Nor agree nor disagree     - Disagree     - Totally disagree     - I do not know   - Chronic pain in an oncological context (for example bone metastases)     - Totally agree     - Agree     - Nor agree nor disagree     - Disagree     - Totally disagree     - I do not know   - Neuropathic pain     - Totally agree     - Agree     - Nor agree nor disagree     - Disagree     - Totally disagree     - I do not know   - Chronic pelvic pain     - Totally agree     - Agree     - Nor agree nor disagree     - Disagree     - Totally disagree     - I do not know   - Post-herpetic pain     - Totally agree     - Agree     - Nor agree nor disagree     - Disagree     - Totally disagree     - I do not know   - Fibromyalgia     - Totally agree     - Agree     - Nor agree nor disagree     - Disagree     - Totally disagree     - I do not know   - Low back pain     - Totally agree     - Agree     - Nor agree nor disagree     - Disagree     - Totally disagree     - I do not know   - Migraine     - Totally agree     - Agree     - Nor agree nor disagree     - Disagree     - Totally disagree     - I do not know   ***Attitude towards complementary medicine in general (not only in the treatment of chronic pain)***   - What impact do these different factors have on your opinion towards the effectiveness of CM therapies?   - Personal experience with positive results on myself:     - Major impact     - High impact     - Moderate impact     - Minimal impact     - No impact     - I do not know   - Recommendations by family and friends who have tried the therapy     - Major impact     - High impact     - Moderate impact     - Minimal impact     - No impact     - I do not know   - Recommendations by colleagues who have tried the therapy themselves     - Major impact     - High impact     - Moderate impact     - Minimal impact     - No impact     - I do not know   - Recommendations by specialists or consultants to whom you have referred a patient     - Major impact     - High impact     - Moderate impact     - Minimal impact     - No impact     - I do not know   - Clinical experience in your patient population     - Major impact     - High impact     - Moderate impact     - Minimal impact     - No impact     - I do not know   - Case report in CM journals     - Major impact     - High impact     - Moderate impact     - Minimal impact     - No impact     - I do not know   - Case report in standard medical journals     - Major impact     - High impact     - Moderate impact     - Minimal impact     - No impact     - I do not know   - Retrospective case–control studies reported in standard medical journals     - Major impact     - High impact     - Moderate impact     - Minimal impact     - No impact     - I do not know   - Prospective randomized controlled clinical trials published in medical journals     - Major impact     - High impact     - Moderate impact     - Minimal impact     - No impact     - I do not know   - Evidence demonstrating the physiological mechanism of CM treatments     - Major impact     - High impact     - Moderate impact     - Minimal impact     - No impact     - I do not know   - Post-graduate training / conferences     - Major impact     - High impact     - Moderate impact     - Minimal impact     - No impact     - I do not know   - Guidelines     - Major impact     - High impact     - Moderate impact     - Minimal impact     - No impact     - I do not know - Who usually starts discussion with a patient about CM therapies?   - Me   - The patients   - I do not know - With approximately which percentage of you patients do you talk about the possible benefits of using CM therapies?   - 0%   - 1-25%   - 26-50%   - 51-75%   - 76-99%   - 100% - With approximately which percentage of your patients do you talk about the possible risks of using CM therapies?   - 0%   - 1-25%   - 26-50%   - 51-75%   - 76-99%   - 100% - Would you recommend a CM therapy to your patients if evidence of its efficiency was available?   - Yes, very favourable   - Yes, favourable   - Neutral   - No, not favourable   - No, not favourable at all - Do you think the introduction of certain CM at the academic center could have an impact on the patients’ satisfaction?   - Yes, very positive impact   - Yes, positive impact   - No effect   - Yes, negative impact   - Yes, very negative impact - Do you think the introduction of certain complementary medicines at the CHUV could attract more patients?   - Totally agree   - Agree   - Neither agree nor disagree   - Disagree   - Totally disagree - Do you think you have enough knowledge about complementary medicines to inform your patients about them?   - Totally agree   - Agree   - Neither agree nor disagree   - Disagree   - Totally disagree - How easy is it for you to find reliable information regarding the use of medical herbs (indications, contraindications, side effects, interactions…) at CHUV?   - Very easy   - Easy   - Neither easy, nor difficult   - Difficult   - Very difficult   - I do not know - How easy is it for you to find reliable information regarding other CM therapies at CHUV (indications, contraindications, side effects, interactions…)?   - Very easy   - Easy   - Neither easy, nor difficult   - Difficult   - Very difficult   - I do not know - Please read and respond to the following statements according to your beliefs:   a) Patients whose physician / nurse / physical therapist / midwife are knowledgeable and practice CM, in addition to conventional medicine, have better clinical outcomes than those whose physicians are only familiar with conventional medicine.   - Strongly disagree - Somewhat disagree - neither agree nor disagree - Somewhat agree - Strongly agree   b) The spiritual beliefs and practices of physicians / nurses / physical therapists / midwives play an important role in the healing of patients.   - Strongly disagree - Somewhat disagree - neither agree nor disagree - Somewhat agree - Strongly agree   c) The spiritual beliefs and practices of patients play an important role in their healing.   - Strongly disagree - Somewhat disagree - neither agree nor disagree - Somewhat agree - Strongly agree   d) Physicians / nurses / physical therapists / midwives should have knowledge about the most prominent CM treatments.   - Strongly disagree - Somewhat disagree - neither agree nor disagree - Somewhat agree - Strongly agree   e) I believe that CM treatments have a true impact on the treatment of symptoms, conditions and/or diseases.   - Strongly disagree - Somewhat disagree - neither agree nor disagree - Somewhat agree - Strongly agree   f) In your opinion, should the CHUV offer scientifically proven CM therapies to its patients?   - Strongly disagree - Somewhat disagree - neither agree nor disagree - Somewhat agree - Strongly agree   g) The introduction of complementary medicines at CHUV could have a negative impact on its reputation:   - Strongly disagree - Somewhat disagree - neither agree nor disagree - Somewhat agree - Strongly agree   h) Complementary medicines are a cost-effective solution:   - - - Totally agree     - Agree     - Neither agree nor disagree     - Disagree     - Totally disagree   i) Healthcare professionals should inform patients about complementary medicines:   - - - Totally agree     - Agree     - Neither agree nor disagree     - Disagree     - Totally disagree   j) More scientific research on complementary medicines is necessary:   - - - Totally agree     - Agree     - Neither agree nor disagree     - Disagree     - Totally disagree   k) I lack information about complementary medicines:   - - - Totally agree     - Agree     - Neither agree nor disagree     - Disagree     - Totally disagree   Have you got any particular expectations from the CHUV regarding complementary medicines?  Commentary on complementary medicines  Commentary on the questionnaire |
| --- | --- |
